# Effect of Irradiation and Detection of Long-Lived Polyenyl Radicals in Highly Crystalline Ultra-High Molar Mass Polyethylene (UHMMPE) Fibers

**DOI:** 10.3390/polym11050924

**Published:** 2019-05-27

**Authors:** Amanda L. Forster, Zois Tsinas, Mohamad Al-Sheikhly

**Affiliations:** 1 Material Measurement Laboratory, National Institute of Standards and Technology, Gaithersburg, MD 20899-8300, USA; 2 Materials Science and Engineering Department, University of Maryland, College Park, MD 20742-2115, USA; ztsinas@umd.edu

**Keywords:** polyethylene, electron-beam irradiation, gamma irradiation, oxidation, crosslinking, electron paramagnetic resonance spectroscopy, high strength fibers, UHMWPE, UHMMPE

## Abstract

To improve properties such as thermal conductivity, low temperature thermal strain, and creep resistance of ultra-high molar mass polyethylene (UHMMPE) fibers, several researchers have previously undertaken efforts to crosslink these fibers using radiation. Ionizing radiation is commonly used to crosslink bulk UHMMPE in other applications, such as artificial joints. However, UHMMPE fibers differ from bulk UHMMPE in that they have a higher crystallinity (approximately 85% to 90%) and are very highly oriented during manufacturing in which the fibers are stretched 50 to 100 times their original length. Thus, the amorphous fraction of the UHMMPE fibers is also highly ordered. Several experiments were conducted to crosslink the UHMMPE fibers using both low dose rate (gamma) and high dose rate (electron beam) irradiation, all in the absence of oxygen. In all cases, the tensile strength of the fiber was greatly reduced by the irradiation. The oxidation index was also measured for the irradiated samples, and oxidation was not found to play a major role in the reduction of tensile strength in the fibers after irradiation. While this work did not achieve the desired result of improving the mechanical properties of the UHMMPE fiber, a significant result was found. The electron paramagnetic resonance (EPR) spectrum of the UHMMPE fibers was measured shortly after irradiation, and a mixture of allyl and alkyl radicals were detected. The irradiated samples were stored in dark ambient conditions for at least six years, then reexamined using EPR for free radical characterization. Surprisingly, the gamma-irradiated samples showed clear evidence of long-lived polyenyl radicals present in the material. Free radicals are very reactive species that will typically migrate to the surface of the crystalline domain and decay in a relatively short time through various reactions in the amorphous regions. It is hypothesized herein that due to the high crystallinity and large anisotropy of the highly drawn UHMMPE fiber, the polyenyl radicals were trapped in the crystal phase and were unable to migrate and decay. An experiment was performed to test this hypothesis, by which samples of the irradiated fibers were heated to temperatures above first the alpha relaxation and then melting point of polyethylene, and EPR measurements were taken. Results showed that the polyenyl radical signal persisted below the *T*_m_, but was rapidly eliminated upon melting of the crystals. These experiments support the hypothesis that the long-lived polyenyl radicals are trapped in the crystalline region of the polyethylene fibers.

## 1. Introduction

**Figure 1:**
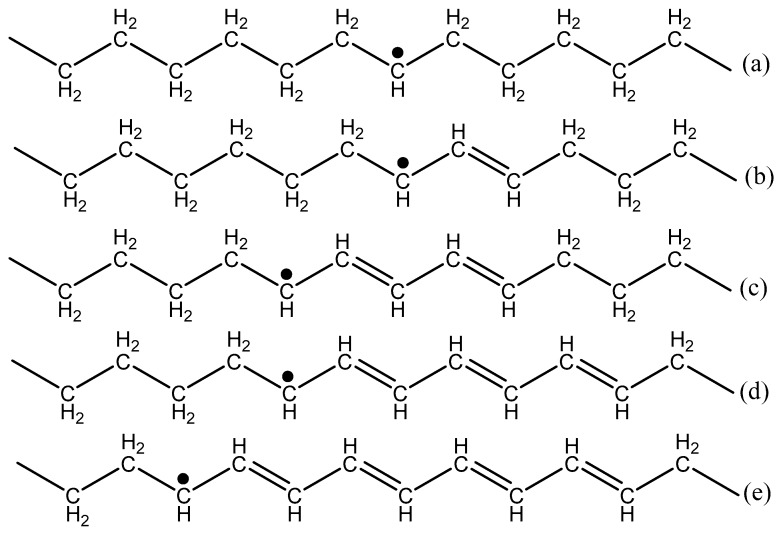
Free radicals expected from the irradiation of polyethylene. Schematic drawing of the (**a**) alkyl, (**b**) allyl, (**c**) dienyl, (**d**) trienyl, and (**e**) tetraenyl free radicals.

To improve properties such as thermal conductivity [1], low temperature thermal strain [2], and creep resistance of ultra-high molar mass polyethylene (UHMMPE) fibers, many researchers have undertaken efforts to crosslink these fibers using radiation [1,3,4,5,6,7,8,9,10,11]. Exposure to ionizing radiation produces alkyl free radicals (Figure 1) in both the crystalline and amorphous regions of the polyethylene, however subsequent reactions of these moieties occur in the amorphous phase. Alkyl free radicals are unstable and can decay via one of three different mechanisms. These are intramolecular recombination, which results in a trans-vinylene unsaturation, intermolecular recombination, which forms a crosslink, or the alkyl radical can migrate to an allylic position of an unsaturation, forming either an allyl free radical if it is a vinylene, or forming a polyenyl free radical if it is a polyene unsaturation [12].

**Figure 2:**
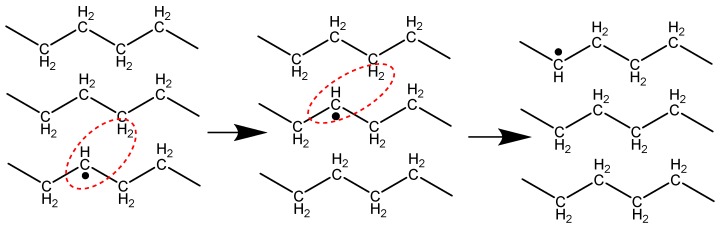
Hydrogen abstraction from one chain to another, and the simultaneous hop of the radical site. Reproduced from [19], with the permission of AIP Publishing. Copyright AIP Publishing, 1987.

Crosslinking in the crystalline region of the polymer is not favored because the spacing between the chains (4.1 Å) is longer than the carbon–carbon bond length (1.5 Å) [13]. In the crystalline region of the polymer, alkyl free radicals are expected to migrate to the crystal surface, where crosslinks can be formed. The primary mechanism by which the radicals can migrate within the crystal is a set of successive inter or intra-molecular hydrogen abstraction reactions, which is also known as hydrogen hopping [14,15,16,17,18]. Through this mechanism, a radical site on a macromolecule abstracts a hydrogen atom from an adjacent carbon atom (either on the same chain or a nearby chain). This process can repeat itself, another hydrogen can be abstracted, and the radical can move once again to a new location, and so on. Figure 2 shows the first step in this hydrogen hopping mechanism [19].

Irradiation of polyethylene can also lead to chain scission, which is a reduction of the average molar mass of a macromolecule [20]. Typically, bulk polyethylene will preferentially form crosslinks during radiation treatment in an inert atmosphere, with the ratio between the number of main chain scissions to crosslinks not exceeding 0.1 [11,19,21]. Irradiation treatments are typically designed to favor crosslinking, however, oxidative chain scission can also occur when oxygen diffuses into the polymer during or after irradiation [21].

Much work has been published on the use of low dose rate gamma irradiation in various doses to crosslink UHMMPE fibers [6,7,8,11,22,23,24,25]. In one such paper, deBoer and Pennings [8] were successful at crosslinking the fibers, however, they observed a marked decrease in tensile strength (up to 40%) with increasing dose. The authors attributed this decrease in tensile strength to preferential chain scission of stressed chains in the fiber structure [8]. Deng, et al. revisited the topic of gamma irradiation of UHMMPE fibers [7]. Samples were exposed to a nominal dose in three environments using a ^60^Co source. Deng’s study showed an increase in tensile strength immediately after irradiation for all samples except for those irradiated in air, but 160 days after irradiation, a major reduction in tensile strength was observed and the authors concluded that gamma irradiation was detrimental to the fibers [7].

Many researchers have examined high dose rate electron beam irradiation as a potential means of crosslinking UHMMPE fibers [4,5,10,26,27]. Klein et al. published a paper in 1987 examining the effectiveness of irradiation of UHMMPE fibers for crosslinking [5]. They noted that most crosslinking occurs in the amorphous regions of the polymer, meaning that samples of low crystallinity are more efficiently crosslinked than those of high crystallinity. Klein also noted that for the case of irradiation of UHMMPE gel spun fibers, chain scission is an important reaction [5], whereas in the case of bulk polyethylene, crosslinking is the dominant reaction and chain scission is relatively unimportant. Klein exposed UHMMPE fibers to radiation, then dissolved the irradiated fibers and measured the resulting gel (insoluble material is an indicator of crosslinking). They reported an increase in gel fraction up to a dose of 1.3 kGy, but dropping significantly at higher doses [5]. This is in direct contrast to polyethylene fibers of lower molecular weight, irradiated in an acetylene environment at doses up to 48 kGy, in which gel content was shown to increase almost linearly with dose and also resulted in an improvement in mechanical properties [26]. This contrast was explored by Dijkstra and Pennings in 1988 [4], who discussed the role of taut-tie molecules (TTM) in the irradiation of UHMMPE fibers. The tensile strength of the UHMMPE fiber depends on the TTM chains that connect crystal blocks in the fiber structure. Stresses in the microfibrils are transferred between crystals by the TTMs and the disordered region between crystals, making these two regions the weakest points in the microfibrils. Upon irradiation of the fiber and subsequent scission of the TTM, both ends of the TTM may immediately recoil due to the sudden release of stress in the chain. The two radicals may be separated over such a distance that the probability of their recombination is unlikely, and tensile stress must be transferred by the remaining TTMs. This theory offers a reasonable explanation of the difficulty encountered in crosslinking UHMMPE fibers, though absent from discussions of this topic is the role of dose rate in chain scission or crosslinking of UHMMPE fibers or any description of free radical chemistry [4]. Only one study by Zhao et al. [27] examined the free radicals formed in the UHMMPE fibers after irradiation. They detected alkyl radicals in the fibers after irradiation, which converted to allyl and then polyenyl radicals. The authors noted the long life of the polyenyl radicals, which were still detected after 137 days of storage, and attributed it to the large crystallite size in the fibers.

As previously shown, irradiation of UHMMPE fibers requires carefully controlled experiments due to the sensitivity of the fibers to chain scission. In the present work, the effect of both low and rapidly pulsed high dose rate radiation in an argon environment on strength, oxidation, and radical formation in UHMMPE fibers is examined immediately after irradiation, and again several years later to understand the effect of long ambient aging times on the free radical species formed in irradiated highly crystalline UHMMPE fibers.

## 2. Experimental

### 2.1. ^60^Co Gamma Irradiation-Low Dose Rate Experiments

Prior to irradiation, commercially available gel spun ultra-high molar mass polyethylene (UHMMPE) filament yarns with a nominal molar mass of 3 to 5 million amu and an approximate tensile strength of 3.8 GPa were wound onto glass cylinders and packaged in aluminized polyethylene. The packages were purged with argon for at least 30 min before heat sealing. The packaged samples were then placed in the ^60^Co gamma source for irradiation. The dose rate obtained for the sample’s position from simulation was 29 kGy/h. Radiochromic thin film dosimeters with a range from 0.5 to 200 kGy were placed on the samples and measured via ultraviolet visible spectroscopy. These films indicated that the dose rate was slightly lower than initially calculated. Based on the time the samples were irradiated, and the measured doses of the samples, the actual nominal dose rate was approximately 25.2 kGy/h. Doses ranged from 8.0 to 86.8 kGy for samples in this experiment, and the intended range was 10 to 100 kGy.

### 2.2. High Dose Rate Irradiation Experiments

Fibers were irradiated under a continuous argon purge using a custom designed apparatus to continuously feed a fiber bundle through the electron beam. Fibers were irradiated using the Medical and Industrial Radiation Facility (MIRF) (Gaithersburg, MD, USA), which is a pulsed electron beam with an energy range that is continuously variable from 7 to 32 MeV. All experiments presented herein were performed with MIRF set to 10 MeV. The pulse width was 6 µs. The pulse repetition frequency was 120 pulses per second (pps). The beam was assumed to be uniform and had a width of approximately 2 cm. Using the scan speed, and the measured dose values from radiochromic film, the dose per second to the UHMMPE fiber can be estimated. The radiochromic film was calibrated by irradiation in a calibrated ^60^Co gamma irradiation source with a dose rate of 47.1 kGy/h at 15 cm from the centerline. Further details of this experiment are available in a previous publication [28].

### 2.3. Free Radical Determination

Electron paramagnetic resonance (EPR) spectroscopy was used to identify the different types of the free radicals in the UHMMPE fibers that were induced by gamma or electron beam irradiation. Some samples were tested immediately after irradiation, and the measurements were carried out at room temperature in an ambient environment. Additionally, some irradiated fiber samples were kept in the lab in a dark, ambient, aerobic environment for approximately 6 years. These samples were then placed in EPR tubes and purged with argon gas for at least 30 min, then immediately sealed with paraffin laboratory film. EPR measurements were taken within a few minutes of sealing to evaluate the presence or absence of free radicals in the fibers. Next, the samples in the same EPR tubes were transferred in oil baths at 80 and 160 °C and were heated for various times, then EPR measurements were performed on all samples at each time point and temperature, within minutes of being removed from the oil bath. The EPR spectra were acquired on an ESP300 spectrometer (Bruker Biospin, Billerica, MA, USA) using the following instrument parameters: microwave frequency of 9.44 GHz, microwave power of 0.5 mW, frequency modulation of 100 kHz, modulation amplitude of 3.12 G, receiver gain of 56,400, center field at 3350 G, a sweep width of 800 G, a conversion time of 20.48 ms, and a time constant of 20.48 ms.

### 2.4. Wide Angle X-ray Scattering

Wide (small) angle X-ray scattering (W(S)AXS) measurements were conducted in transmission using a Xenocs Xeuss SAXS/WAXS small angle X-ray scattering system (Sassenage, France). The instrument was equipped with an X-ray video-rate imager for SAXS analysis with a minimum Q = 0.0045 Å^−1^ and a separate X-ray video-rate imager detector for WAXS analysis (up to about 45° 2θ). All diffractograms were collected at room temperature. The incident beam, diffracted beam and sample chamber were kept under vacuum. The bundle of UHMMPE fibers was mounted horizontally, perpendicular to the direction of the X-ray beam. Silver behenate was used as a control and each sample was tested in duplicate. Further details of the same experimental setup are described in a previous paper [29].

### 2.5. Measurement of Melting Points and Crystallinity

Irradiated and unirradiated UHMMPE fiber samples were characterized by differential scanning calorimetry (DSC), Fourier transform infrared analysis (FTIR), and tensile testing. A Q2000 DSC (TA Instruments, New Castle, DE, USA) with a refrigerated cooling system (RCS) was used to measure the crystalline melting temperatures and the percent crystallinity for all UHMMPE fiber samples. Samples ranging from 3 to 5 mg were cut and coiled in the bottom of a non-hermetically sealed aluminum sample pan. Experiments were performed in standard mode with a nitrogen purge. All samples were heated from 0 to 170 °C, then cooled to 0 °C, then heated back to 170 °C in a standard heat–cool–heat experiment. The heating and cooling rates were 5 °C/min. Percent crystallinity was calculated using the standard enthalpy of pure crystalline polyethylene as 291 J/g [30].

### 2.6. Oxidation Analysis

Infrared analysis was carried out using a Bruker Vertex 80 FTIR (Billerica, MA, USA), equipped with a Smiths Detection Durascope Attenuated Total Reflectance (ATR) accessory (London, UK). Nitrogen was used as the purge gas. Consistent pressure on the yarns was applied using the force monitor on the Durascope. FTIR spectra were recorded at a resolution of 4 cm^−1^ between 4000 and 700 cm^−1^ and averaged over 128 scans. Standard uncertainties associated with this measurement are typically 4 cm^−1^ in wavenumber and approximately 5% in peak intensity. Three different locations on each yarn were analyzed.

### 2.7. Mechanical Properties Measurement

Tensile testing of yarns was carried out in accordance with ASTM D2256-02, “Standard Test Method for Tensile Properties of Yarn by the Single-Strand Method” [31], using an Instron Model 4482 test frame equipped with a 91 kg (200 lb) load cell, and pneumatic yarn and cord grips (Instron model 2714-006). The jaw separation was 25 cm (9.8 in) and the cross-head speed was 25 cm/min (9.8 in/min). In this study, yarns were nominally 58.4 cm (23 in) long and given 23 twists on a custom designed yarn twisting device. This level of twist was maintained on the yarns as they were inserted into the pneumatic yarn and cord grips. Strain measurements were made with an Instron non-contacting Type 3 video extensometer in conjunction with black foam markers placed approximately 2.5 cm apart in the gauge section of the yarn. Ten to twelve replicates from each sample set were tested to failure. The standard uncertainty of these measurements was typically 5%.

### 2.8. Dynamic Mechanical Thermal Analysis

Dynamic mechanical thermal analysis (DMTA) was performed using a TA Instruments RSA III (New Castle, DE, USA) to examine the thermomechanical properties of the fibers. A single fiber samples were mounted in the instrument using film and fiber grips and measured using a temperature-frequency sweep from 30 to 110 °C, at a frequency of 1 Hz. At least 3 replicates of each sample were tested. Standard uncertainties associated with this technique are approximately 5% in modulus and 1% in temperature.

## 3. Results and Discussion

### 3.1. Determination of the Presence of Free Radicals in Irradiated Fibers

**Figure 3:**
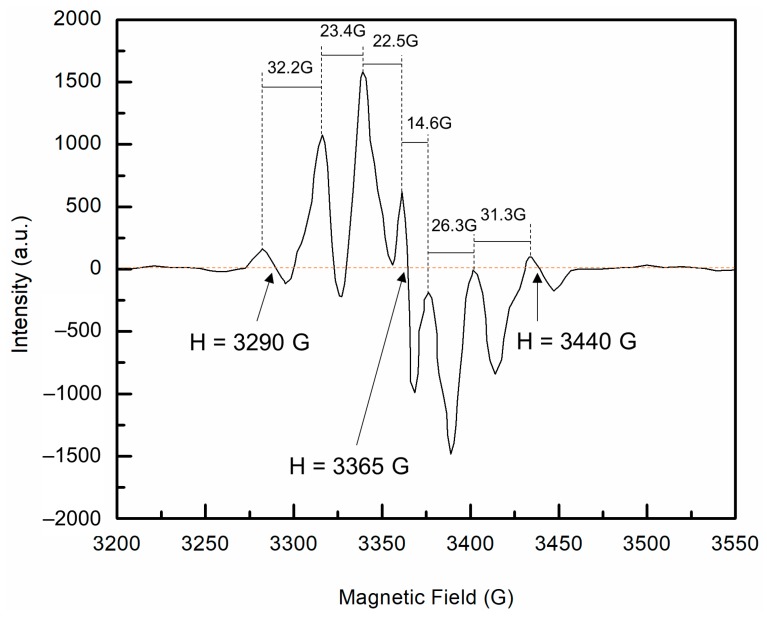
Electron paramagnetic resonance (EPR) spectra of ultra-high molar mass polyethylene (UHMMPE) fibers irradiated to approximately 25 kGy in the electron beam. The first derivative is a septet attributed to the combination of allyl and alkyl radicals in the sample. The separation between the lines is shown including the magnetic field positions of the first and seventh line, H = 3290 G and H = 3440 G respectively, as well as the center of resonance H = 3365 G.

Samples of UHMMPE fiber irradiated to approximately 25 kGy using the electron beam were examined using EPR, as described previously for unirradiated samples. There are three common radicals in irradiated polyethylene: the alkyl, the allyl, and the polyenyl free radicals. The alkyl free radicals are commonly studied, but can be difficult to detect because they have a very short lifetime and rapidly convert to the allyl radicals. Figure 3 shows the EPR spectrum of UHMMPE fiber samples irradiated in the electron beam. The septet depicted in this figure is a combined spectrum of the allyl and the alkyl radicals found in irradiated polyethylene [12,32,33]. A complex EPR spectrum containing a combination of allyl and alkyl radicals was also previously detected by Zhao et al. in irradiated UHMMPE fibers [27].

### 3.2. Oxidation Determination in Irradiated UHMMPE Fibers

**Figure 4:**
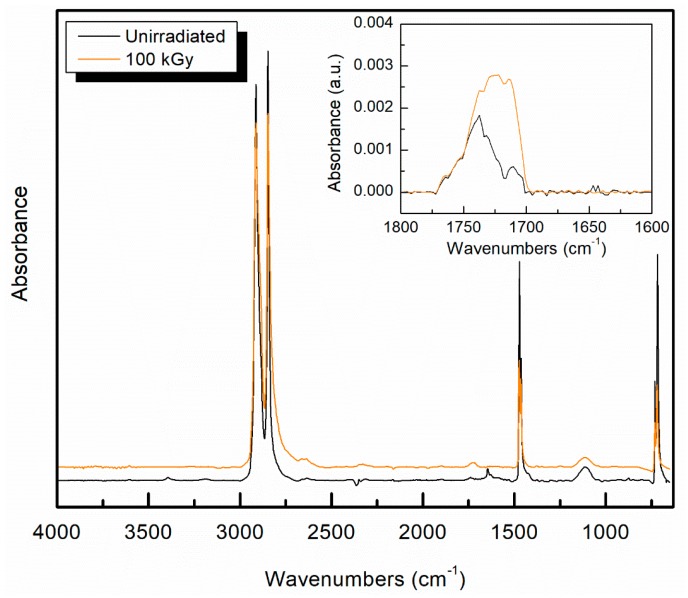
The complete FTIR spectra of unirradiated and gamma irradiated UHMMPE fibers at 100 kGy; inset shows the region in which oxidation would be expected; the two IR spectra have been slightly offset to allow a clearer presentation.

There are four characteristic infrared peaks for UHMMPE, identified as C–H stretching at 2914 and 2846 cm^−1^, and C–H bending at 1471 and 1462 cm^−1^. Figure 4 shows the complete FTIR spectra of unirradiated fibers and gamma irradiated fibers at 100 kGy, as well as the corresponding oxidation peaks between 1800 and 1600 cm^−1^ as an inset.

**Figure 5:**
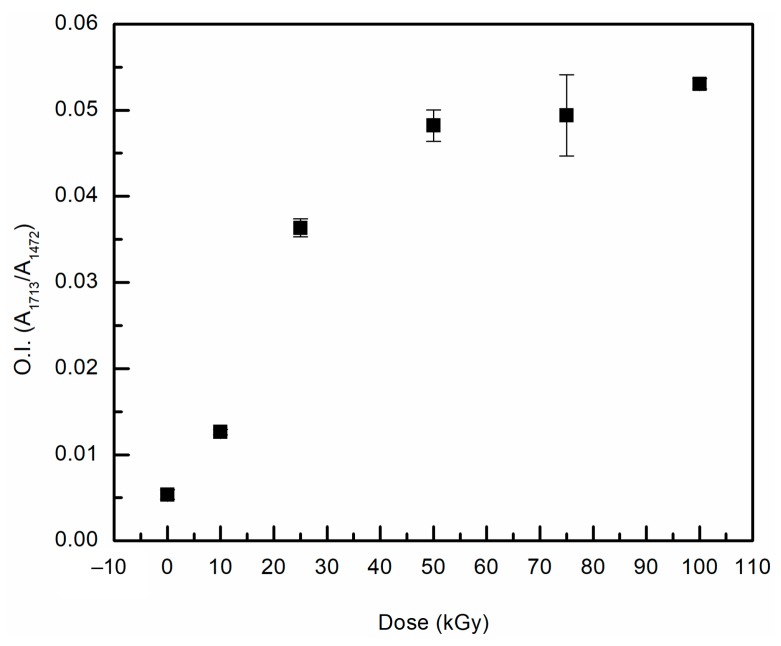
Oxidation index for all gamma irradiated UHMMPE fibers at different doses.

Oxidation is typically measured using FTIR for UHMMPE fibers from peaks in a range between 1689 and 1756 cm^−1^, and typically focuses on a peak at 1740 cm^−1^ [34,35]. To examine the samples for evidence of oxidation, the oxidation index [36] was calculated and plotted against the total dose, as shown in Figure 5. The data show a statistically significant (Student’s t-test, *p* ≤ 0.05) increase in the oxidation index of irradiated fibers as compared to the unirradiated samples. A noticeable increase in the oxidation index of the fibers was observed at doses of 25 kGy and above. Also, at total doses of 50 kGy and above the changes in the oxidation index was within statistical error, indicating that oxidation of the fibers plateaus at high doses. This may be because the fibers were purged with argon prior to irradiation, so only the oxygen that was dissolved in the polymer is available for oxidation reactions. At some point, the available oxygen may have been consumed and further oxidation of the fiber may not be possible. These samples are commercial materials and have been previously determined to contain an antioxidant, which does impact free radical reactions within the material [36]. Antioxidants trap and deactivate free radicals in the polymer, thus preventing oxidation reactions from occurring. This is a desirable effect in most applications, however, it can present problems during crosslinking with irradiation. In this case, free radicals are essential for the crosslinking to occur. If these radicals are trapped and deactivated by the antioxidant, there are fewer radicals available to participate in crosslinking. The presence of antioxidant in the material will also impact the oxidative aging of the material over time, extending the time in which the material can be stored in air without undergoing detrimental oxidation [36,37,38].

### 3.3. Effect of Radiation on Crystallinity of UHMMPE Fibers

**Figure 6:**
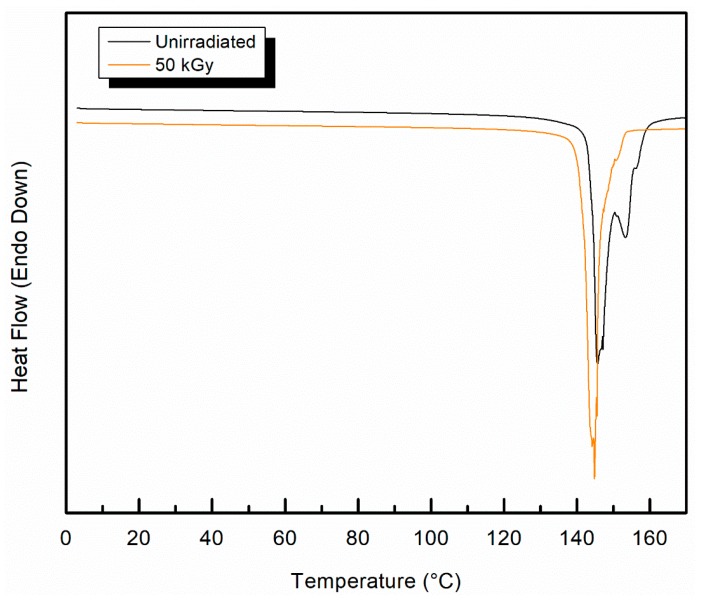
Differential scanning calorimetry (DSC) thermogram for virgin and gamma irradiated polyethylene, 1st melt; the two thermograms have been slightly offset to allow a clearer presentation.

Representative DSC thermograms for the first melting of the sample irradiated at 50 kGy using gamma irradiation overlaid with a thermogram of the first melting of an unirradiated sample, are shown in Figure 6. All samples tested exhibited broad and complex melting peaks due to a phase transformation of the crystal from the orthorhombic to the hexagonal phase, which is characteristic of this material, and has been extensively reported elsewhere [39,40,41]. The primary melting peak at 149 °C, which is typically indicative of the melting of the orthorhombic crystal phase, was still present after irradiation [42]. However, minor changes are observed in the thermogram. The sample exhibited a lower onset of melting temperature, a narrower melting peak, and a near elimination of the minor, higher temperature, melting peak. The percent crystallinity was unchanged with irradiation, estimated at 91% for the unirradiated fiber and 90% for the irradiated fiber. This difference is within the error of the measurement.

**Figure 7:**
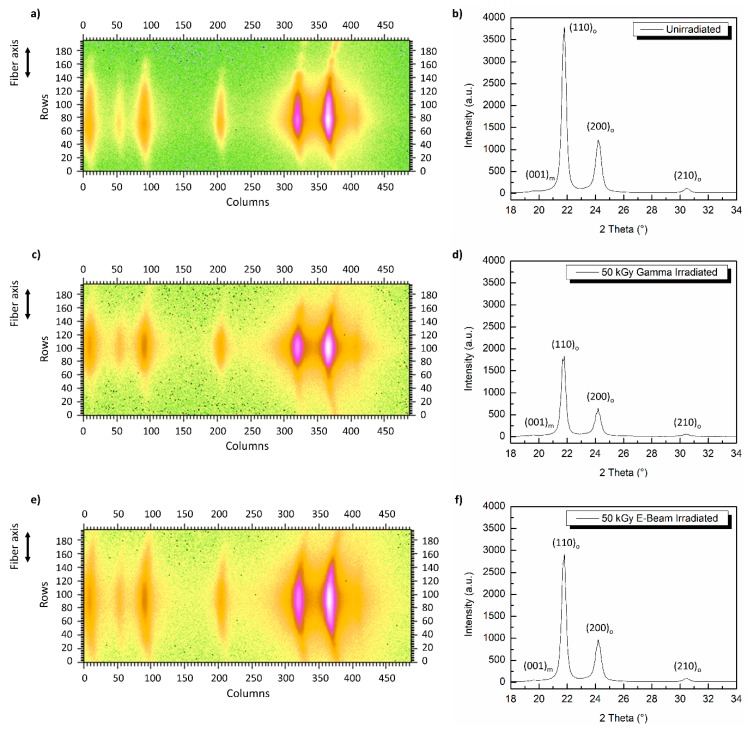
Two-dimensional wide angle X-ray scattering (WAXS) patterns and their corresponding diffractograms for UHMMPE fibers; (**a**,**b**) untreated, (**c**,**d**) 50 kGy gamma irradiated, (**e**,**f**) 50 kGy electron beam irradiated.

Wide angle X-ray analysis of the fibers was conducted in parallel with other measurements to assess the changes in the crystallinity of the irradiated fibers. The equatorial 2D WAXS pattern and diffractogram of virgin UHMMPE fibers are shown in Figure 7a,b respectively. The results revealed the presence of two strongly diffracting peaks at 2θ angles around 21.5° and 23.5° corresponding to the (110)_o_ and (200)_o_ lattice planes of the orthorhombic crystals [40,43,44,45]. Also, two weak diffraction peaks were present, one at 2θ ~19.5° corresponding to the (001)_m_ lattice plane of the monoclinic crystal phase and one at 2θ ~31.5° associated to the (210)_o_ plane of the orthorhombic crystals [40,43,44,45]. The orthorhombic crystal phase is expected to comprise the core of the fiber, and a small fraction of the monoclinic phase has been reported to be found in the fiber sheath, due to the mechanical stress of extrusion and drawing [46,47]. While these measurements confirm the presence of a monoclinic phase, the instrument used for this measurement was not able to quantify the amount or location of the monoclinic phase. After the irradiation of these fibers using electron-beam (Figure 7e,f) or gamma-rays (Figure 7c,d) at a total dose of ~50 kGy, no major changes were observed on their crystal structure. The wide angle diffractograms revealed the presence of the same four peaks, (001)_m_, (110)_o_, (200)_o_ and (210)_o_ as in the case of the virgin fibers. Thus, the monoclinic and orthorhombic crystals of these fibers were still present and not affected significantly by the above irradiation conditions.

### 3.4. Effect of Radiation on the Mechanical Properties of UHMMPE Fibers

**Figure 8:**
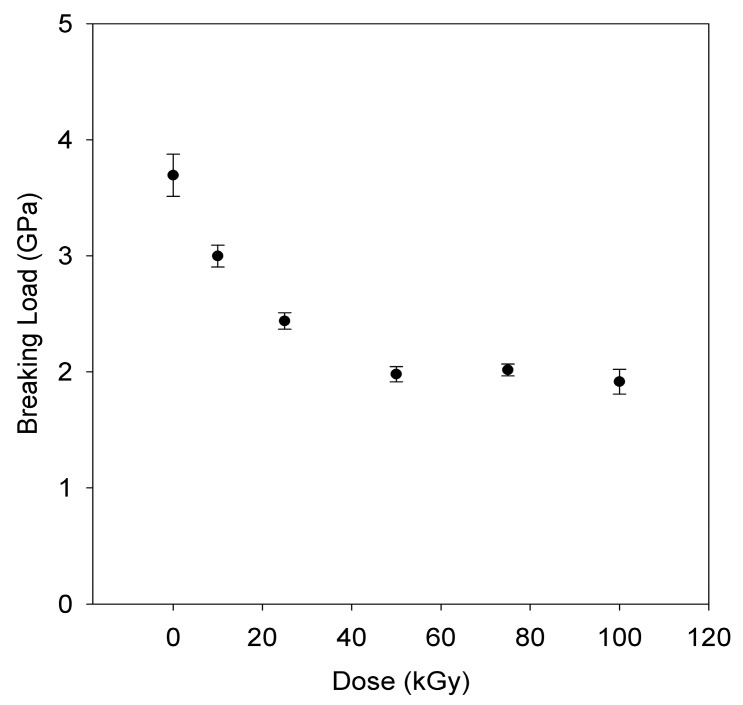
Breaking strength of irradiated UHMMPE fibers as a function of dose.

Breaking strength vs. dose for the gamma irradiated fibers are presented in Figure 8, and breaking elongation followed a similar trend. This reduction in strength is attributed to the scission of TTMs in the fiber during irradiation. At doses exceeding 50 kGy, the reduction was diminished, indicating that most of the available sites for scission appear to have been exhausted.

Several experiments were conducted to evaluate the effect of different doses on the fiber. In all cases, the tensile strength was greatly reduced by the irradiation. The tensile strength results are presented in Table 1. The sample with a speed and dose of zero is the unirradiated control fiber.

The polymer chains may still have been preferentially scissioning, as was previously observed in the gamma irradiation experiments. This may be because carbon centered free radicals were not generated fast enough to crosslink the TTMs before they scissioned due to the limitations of the pulse rate of the source. Even the very low dose sample exposed to 8 kGy showed a significant reduction in tensile strength (32%). The mechanical properties of polymers in the solid state are influenced strongly by molecular motions under the given thermodynamic condition and applied mechanical stress. The temperature at which a molecular motion stops or starts is known as a relaxation or transition temperature. This relaxation intensifies, meaning that the relative increase in tan delta (the ratio of the loss modulus to the storage modulus) is greater, with increasing crystallinity, so it usually assigned to the motion of chain units within the crystalline region [48]. In polyethylene, the α transition is well known, and expected to occur in the region around 70 °C. This relaxation is important for many applications of UHMMPE fibers because it is apparent as a loss in strength of the UHMMPE fiber. Previous work has shown that radiation crosslinking of polyethylene can reduce the magnitude of the α relaxation [49]. The samples irradiated using the electron beam at 3 cm/s were also analyzed using dynamic mechanical thermal analysis (DMTA) to interrogate the susceptibility of UHMMPE fibers to changes in their properties over their potential range of use temperatures due to their α relaxation. All four replicates of the irradiated sample broke during the experiment.

**Figure 9:**
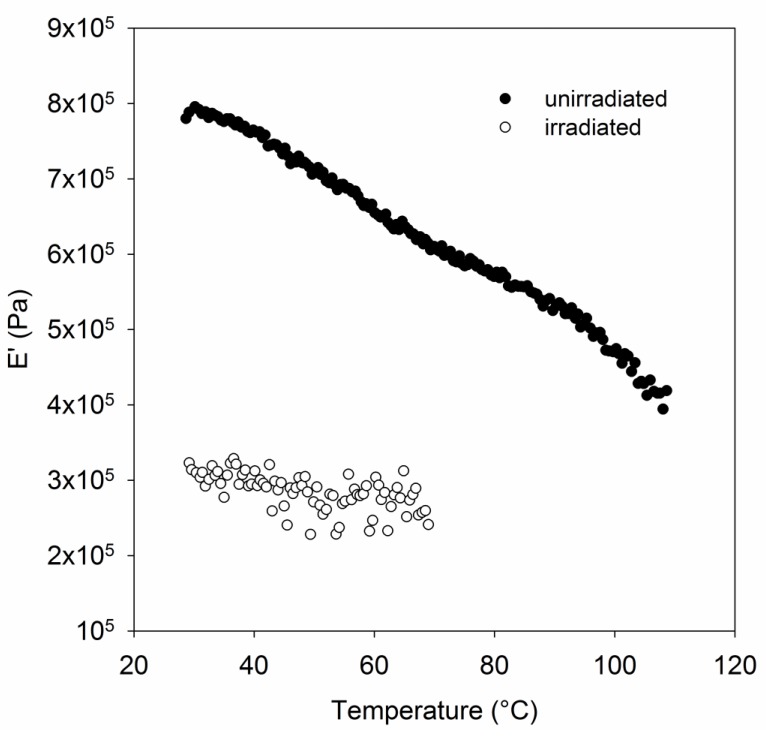
Dynamic mechanical thermal analysis (DMTA) temperature–frequency sweep of irradiated and unirradiated UHMMPE fiber.

A representative plot for an unirradiated sample is shown in Figure 9 overlaid with data for the irradiated sample, which broke around 80 °C. The weakening of the irradiated fiber by the treatment is apparent from this analysis, however the temperature dependence of the sample appears to be much less sensitive than the unirradiated sample, so irradiation treatment may have crosslinked the fiber.

### 3.5. Detection of Extremely Long-Lived Polyenyl Radicals

**Figure 10:**
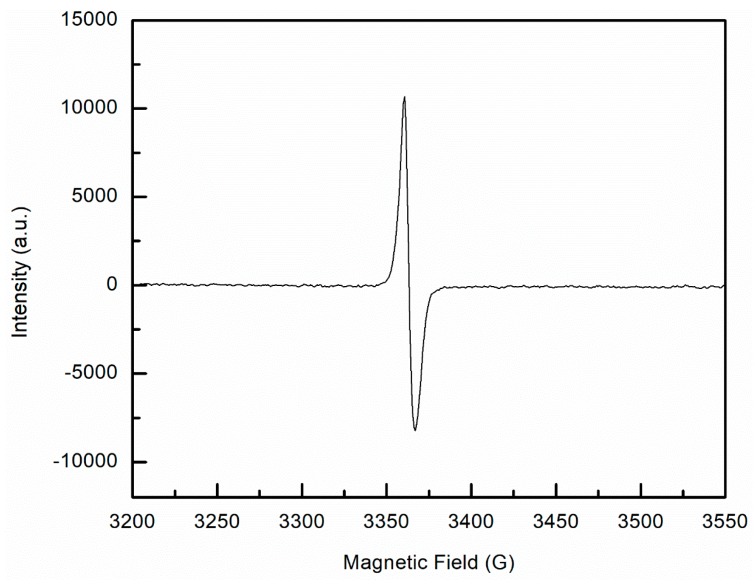
EPR spectrum of present polyenyl free radicals in 50 kGy gamma irradiated UHMMPE fibers six years post irradiation.

The work described herein has shown that this UHMMPE fiber exhibits similar behavior when exposed to radiation as do other types of gel-spun UHMMPE fibers—primarily a reduction in tensile strength, which may be due to chain scission. While this work did not achieve the desired result of improving the mechanical properties of the UHMMPE fiber, one notable result was determined. The irradiated fibers were tested as described previously and stored in dark ambient conditions for approximately six years, then reexamined using EPR for free radicals. Surprisingly, the gamma-irradiated samples showed clear evidence of long-lived polyenyl radicals, as shown by the strong singlet in Figure 10, which was irradiated under argon at 50 kGy and stored in an aerobic, dark ambient environment.

One possible explanation for the presence of these polyenyl radicals so long after irradiation is the high crystallinity of these UHMMPE fibers (approximately 85%) as compared to typical bulk polyethylene, which is typically around 75% crystalline [50]. The alkyl radicals formed immediately after irradiation would react quickly to form polyenyl radicals. As previously described, these polyenyl radicals would migrate around the crystalline region via hydrogen hopping. Typically, these polyenyl radicals would be expected to eventually migrate to the surface of the crystalline domain and be eliminated in the amorphous region. However, we suspect that due to the high crystallinity and large anisotropy of the highly drawn UHMMPE fiber, the polyenyl radicals were unable to be eliminated and were trapped in the crystal. An experiment was performed to test this hypothesis, which will be described in the next section.

### 3.6. Effect of Temperatures above the Alpha Relaxation on Long-Lived Polyenyl Radicals

A sample of the long-aged irradiated UHMMPE fiber was measured in the EPR, and the polyenyl radical signal was detected. Then, the sample was purged for at least 15 min with argon and sealed tightly with paraffin wax film. The EPR tube was immersed in an oil bath maintained at 80 °C for 5, 15, 30, and 60 min, and then was tested to assess the amount of free radicals present. The alpha relaxation temperature of 80 °C was selected for this experiment to increase the molecular motion within the polymer without melting the crystals. If the polyenyl radicals were in the amorphous regions of the polymer, then increasing the molecular motion should increase the probability of the polyenyl radicals to recombine and eliminate. However, if the polyenyl radicals were trapped in the crystalline regions of the polymer, then they would be expected to remain. This experiment was repeated several times, and only slight changes in intensity of the polyenyl radical (likely due to different positioning of the sample within the EPR chamber) were observed.

**Figure 11:**
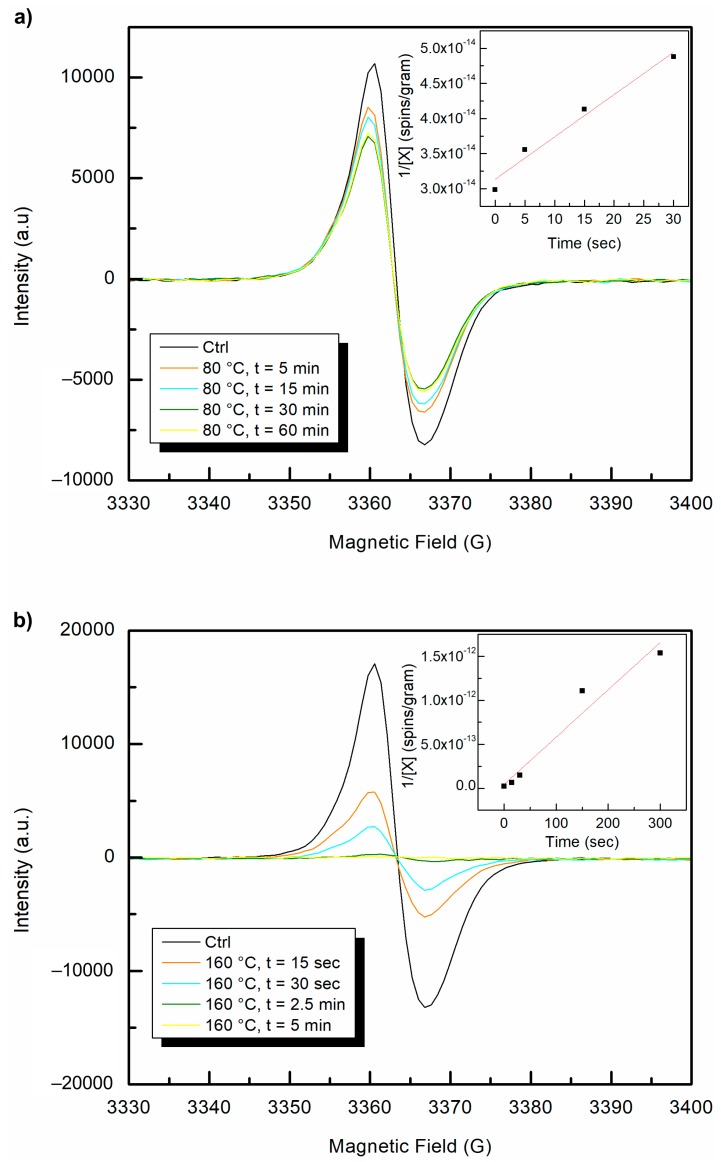
EPR polyenyl free radical spectra of UHMMPE fibers six years after gamma irradiation at 50 kGy. Post irradiation heat treatment at (**a**) 80 °C and (**b**) 160 °C and the kinetics of free radical decay. Inset plots show the change in spins per gram as a function of heating time.

Once it was clear that the polyenyl radicals remained at temperatures above the alpha-relaxation temperature, the oil bath was further heated to 165 °C, well above the expected melting temperature for this polyethylene sample [28]. The fibers started to melt immediately but remained in the same position within the EPR tube. The sample was removed from the bath and the EPR signal was remeasured after 15, 30, 150, and 300 s intervals. There were immediate and clear differences in the intensity of the polyenyl radical signal, as shown in Figure 10. The polyenyl radical signal completely disappeared after 2.5 min at 165 °C. To confirm these results, the experiment was conducted thrice using samples of the 50 kGy gamma irradiated polyethylene fibers. In all cases, the polyenyl radicals were eliminated rapidly upon heating above the melting temperature. The decay of carbon centered polyenyl free radicals at both temperatures revealed second order kinetics, as can be seen from the inset graphs shown in Figure 11. This finding was expected since these carbon centered radicals, when under inert conditions, can be eliminated by a radical–radical bimolecular recombination reaction, which is a well-known second order reaction. Finally, the observed reaction rate constant (k_obs_) of the recombination reaction, calculated by the slope, was by an order of magnitude greater at 165 °C (slope value = 5.38 × 10^−15^ or k_obs_ = 8.63 × 10^−36^ mol L^−1^ s^−1^) as compared to the 80 °C (slope value = 6.04 × 10^−16^ or k_obs_ = 9.73 × 10^−37^ mol L^−1^ s^−1^). This result is expected since the mobility of the polymer chains and C-centered radicals in the melt is much greater.

## 4. Conclusions

The results of this study have demonstrated that highly crystalline UHMMPE fiber is very sensitive to irradiation. The primary effects of irradiating the fiber is a reduction in tensile strength. This could be due to chain scission within the fiber. At radiation doses exceeding 50 kGy, the loss of tensile strength appears to diminish, which may indicate that most of the available sites for scission have been exhausted. The same trend is observed in the oxidation index data, in which the increase in oxidation index is reduced at doses above 50 kGy. This may indicate that dissolved oxygen within the material plays a role in the chain scission, since the fibers were all irradiated under argon. DMTA results indicate that while the fiber was weakened significantly by the radiation treatment, some crosslinking may have occurred. Future work will attempt to measure the molar mass of the irradiated fibers to confirm that chain scission occurred.

Very long-lived polyenyl radicals were observed in the irradiated UHMMPE fibers even after approximately six years of storage in aerobic conditions. Experiments conducted at 80 °C (above the alpha relaxation temperature) to eliminate the polyenyl radicals presumed to be in the non-crystalline regions of the polymer revealed some changes in intensity, but did not fully eliminate the free radicals. However, exposure to temperatures above the melting point of the polymer (165 °C) did fully eliminate the polyenyl radicals trapped within the crystalline regions of the material. The elimination of the polyenyl radicals in this system is expected to follow a radical–radical bimolecular recombination mechanism, which is a well-known second order reaction. The radical elimination experiments both followed second order kinetics, and the observed rate constants for both reactions were determined (k_obs_ = 8.63 × 10^−36^ mol L^−1^ s^−1^ at 165 °C and k_obs_ = 9.73 × 10^−37^ mol L^−1^ s^−1^ at 80 °C). These results may inform future models of the distribution of crystalline and non-crystalline regions within the UHMMPE fiber, as the long polyenyl radical lifetime within the fiber further underscores the highly ordered crystal structure of the material.

## Figures and Tables

**Table 1:** The effect of irradiation on tensile strength for UHMMPE fibers irradiated using the electron beam.

Programmed Speed (cm/s)	Estimated Dose (kGy)	Tensile Strength (GPa)	Standard Deviation (kGy)	Percent Reduction (%)
0	0	3.81	0.18	0
0.5	50	2.31	0.17	40
0.75	30	2.37	0.26	38
1.0	25	2.75	0.31	28
3.0	8	2.59	0.52	32
